# Solar Radiation during Rewarming from Torpor in Elephant Shrews: Supplementation or Substitution of Endogenous Heat Production?

**DOI:** 10.1371/journal.pone.0120442

**Published:** 2015-04-08

**Authors:** Michelle L. Thompson, Nomakwezi Mzilikazi, Nigel C. Bennett, Andrew E. McKechnie

**Affiliations:** 1 Mammal Research Institute, Department of Zoology and Entomology, University of Pretoria, Private Bag X20, Hatfield 0028, South Africa; 2 South African Research Chair for Mammal Behavioural Ecology and Physiology, Department of Zoology and Entomology, University of Pretoria, Private Bag X20, Hatfield 0028, South Africa; Universidad de la Republica, URUGUAY

## Abstract

Many small mammals bask in the sun during rewarming from heterothermy, but the implications of this behaviour for their energy balance remain little understood. Specifically, it remains unclear whether solar radiation supplements endogenous metabolic thermogenesis (i.e., rewarming occurs through the additive effects of internally-produced and external heat), or whether solar radiation reduces the energy required to rewarm by substituting (i.e, replacing) metabolic heat production. To address this question, we examined patterns of torpor and rewarming rates in eastern rock elephant shrews (*Elephantulus myurus*) housed in outdoor cages with access to either natural levels of solar radiation or levels that were experimentally reduced by means of shade cloth. We also tested whether acclimation to solar radiation availability was manifested via phenotypic flexibility in basal metabolic rate (BMR), non-shivering thermogenesis (NST) capacity and/or summit metabolism (M_sum_). Rewarming rates varied significantly among treatments, with elephant shrews experiencing natural solar radiation levels rewarming faster than conspecifics experiencing solar radiation levels equivalent to approximately 20% or 40% of natural levels. BMR differed significantly between individuals experiencing natural levels of solar radiation and conspecifics experiencing approximately 20% of natural levels, but no between-treatment difference was evident for NST capacity or M_sum_. The positive relationship between solar radiation availability and rewarming rate, together with the absence of acclimation in maximum non-shivering and total heat production capacities, suggests that under the conditions of this study solar radiation supplemented rather than substituted metabolic thermogenesis as a source of heat during rewarming from heterothermy.

## Introduction

Many mammals offset the energetic cost of continuous endothermic homeothermy through facultative, reversible reductions in body temperature (*T*_b_) and metabolic rate. During bouts of heterothermy, mammalian metabolic rates may be reduced to less than 1% of normothermic values [1]. However, the overall energy savings that mammals achieve through these reductions in metabolic rate are constrained by the energy required to increase *T*_b_ back to normothermic levels at the end of a bout [2, 3]. The rewarming phase typically accounts for most of the energy expenditure during a single bout, and in seasonal hibernators periodic rewarming may represent as much as 80–90% of total energy expenditure [4–7].

Solar radiation represents a practically infinite source of thermal energy to animals, particularly in arid tropical and subtropical regions. Over the last few decades, evidence has accumulated that some species, particularly small marsupials and afrotherians, routinely bask during rewarming from torpor [3, 8–10]. Whereas some of the evidence for basking during rewarming is indirect and based on comparisons between the rewarming rates of animals and those of black bodies (e.g., [3]), direct observational evidence has been presented for dasyurid marsupials [8, 10]. In these studies, animals were documented moving into sunlit sites while their *T*_b_s are still far below normothermic levels (as low as 14.6°C in the case of *Sminthopsis crassicaudata*, [10]).

Despite burgeoning evidence that solar radiation can significantly enhance heat gain in small mammals rewarming from heterothermy, the qualitative and quantitative ways in which basking behaviour influences the energy budgets of free-ranging individuals remains unclear. McKechnie and Wolf [11] noted that the interaction between metabolic thermogenesis and solar heat gain during rewarming from heterothermy can be thought of as a continuum from passive rewarming, where solar radiation substitutes endogenous heat production, to augmented rewarming, where solar heat gain supplements metabolic thermogenesis. Theoretical models and empirical data suggest rewarming rates should vary substantially depending on the mode of rewarming employed. Maximum passive rewarming rates predicted for birds are considerably lower than observed endogenous rates except under conditions of high solar irradiance (> 1000 W m^-2^) and body mass (> 100 g) [11], and rewarming rates inferred as being associated with basking behaviour in free-ranging elephant shrews were much lower than those associated with endogenous rewarming [3]. On the other hand, rewarming rates during basking in several species of dasyurid marsupials appear to be higher than those associated with rewarming in the absence of solar radiation [8, 10]. In laboratory studies, energy expenditure during rewarming is greatly reduced when animals have access to a radiant heat source [12, 13].

We investigated the functional significance of solar radiation during rewarming from torpor in a model afrotherian heterotherm, the eastern rock elephant shrew (*Elephantulus myurus*), by experimentally manipulating the solar radiation available to individuals housed outdoors and experiencing natural cycles of air temperature (*T*_a_). We tested several predictions. First, if solar radiation substitutes for endogenous metabolic thermogenesis, then rewarming rates should either be lower or similar, but not higher, in individuals with access to normal levels of solar radiation compared to individuals that have to rewarm primarily using endogenous heat sources. On the other hand, if solar radiation supplements endogenous heat production (i.e., rewarming rates reflect the additive effect of endogenous thermogenesis and solar heat gain), then rewarming rates should be directly related to the availability of solar radiation and hence higher in unshaded conditions.

We also speculated that the functional role of solar radiation might be manifested through phenotypic flexibility in heat production machinery. There is considerable evidence for acclimation and acclimatization in mammalian minimum and maximum resting metabolic rates [basal metabolic rate (BMR) and summit metabolism (*M*_sum_), respectively] as well as the capacity for non-shivering thermogenesis (NST) [14–18]. The emerging picture of these traits is that they are highly flexible and responsive to environmental factors. Moreover, among some rodents, *M*_sum_/BMR (or metabolic expansibility, ME) is negatively correlated with minimum *T*_b_ during torpor at an interspecific level [19]. The latter evidence for a link between heat production capacity and the characteristics of torpor bouts, taken together with phenotypic flexibility in mammalian *M*_sum_, BMR and NST capacity, raises the possibility that these metabolic variables are adjusted in response to changes in the heat production demands associated with rewarming from heterothermy. We thus predicted that, if solar radiation substitutes for endogenous heat production, then elephant shrews in an unshaded treatment should show reduced heat production capacity compared to those in artificially shaded enclosures. On the other hand, if solar radiation supplements endogenous metabolic thermogenesis, then the heat production capacity of individuals from shaded and unshaded treatments should not differ.

## Materials and Methods

### Study animals and housing

Eastern rock elephant shrews were captured at Welgevonden Private Game Reserve (24° 18'S, 27° 53'E) in the Limpopo province of South Africa, using Sherman traps baited with a mix of oats, peanut butter and sardines, before being transported by road to the University of Pretoria (UP). Mean body mass (*M*_b_) at capture was 56.4 ± 6.8 g. At UP, elephant shrews were initially housed indoors at a constant air temperature (*T*_a_) of 18°C for two weeks to allow them to acclimate to captivity. During this time, elephant shrews were housed individually in 22 x 22 x 23 cm cages with sawdust bedding. Water and a diet consisting of a mash of dog food and ProNutro breakfast cereal with daily supplements of an apple and carrot mixture, as well as mealworms once a week, was provided *ad libitum* during this initial period. Average food intake was estimated by weighing food bowls before and after feeding each day.

Following the two weeks of indoor housing, animals were transferred to an open field at the UP Experimental Farm (25° 45'S, 28° 15'E) where they were housed individually in 1.2 x 1.2 x 1.2 m wire mesh cages, each with a rock pile intended to mimic their natural habitat. Water was provided *ad libitum*, and a quantity of food equivalent to 70% of the mean *ad libitum* consumption during the preceding period of indoor housing was provided each day.

Animals were captured with permission from Limpopo Department of Economic Development, Environment and Tourism (001-CPM402-00001). All procedures were approved by the University of Pretoria’s Animal Ethics Committee (EC-028-12).

### Body and air temperature measurements

For experiments involving the manipulation of solar radiation availability, body temperatures were measured using surgically implanted miniature temperature loggers (Thermochron iButton, model 1922L, Maxim Dallas Semiconductors, Sunnyvale, CA, USA). An iButton was implanted into the intraperitoneal cavity of each elephant shrew under inhalation anaesthesia by a veterinarian, following two weeks of indoor housing. This procedure took place a week before data collection commenced, allowing animals to recover fully. The iButtons weigh less than 5% of the elephant shrews’ *M*_b_, and do not negatively influence activity or behavioural patterns in this species [3, 20]. The iButtons recorded *T*_b_ at 15-min intervals for a period of 42 days. Prior to implantation, all iButtons were calibrated over a temperature range of 0–45ºC in a circulating water bath against a mercury-in-glass thermometer with NIST-traceable accuracy.

Air temperature, solar radiation (W m^-2^) and other weather variables were recorded using a portable weather station (Vantage Pro2, Davis Instruments, Haywood, CA, USA) placed in the same area as the cages. The air temperature sensor of this weather station had previously been calibrated against a mercury-in-glass thermometer in a controlled-temperature cabinet.

For laboratory measurements of heat production capacity (which involved the same individuals following the end of the outdoor solar radiation availability experiments), *T*_b_ was measured using temperature-sensitive passive integrated transponder (PIT) tags (Destron Fearing, St. Paul, MN, USA) injected subcutaneously between the scapulae of each elephant shrew. The PIT tags allowed *T*_b_ to be measured using a racket antenna (Racket Antenna, Biomark, Boise, Idaho, USA) attached to a PIT tag reader (Model FS2001F-ISO, Biomark, Boise, Idaho, USA). Air temperature within each chamber used for metabolic measurements (see below) was measured using a thermistor probe (Sable Systems, Las Vegas NV, USA) inserted through a small hole in the chamber wall and sealed with a rubber grommet.

### Experimental manipulation of solar radiation availability

In order to investigate the influence of solar radiation on patterns of heterothermy and rewarming rates, we allocated elephant shrews to one of three experimental treatments, namely full sun (n = 5), partial shade (n = 6) or deep shade (n = 7). Allocations were randomized, but sex ratios were kept approximately equal in each treatment. We varied solar radiation (SR) availability by attaching shade cloth to a ~ 2.6 m x 1.5 m frame constructed of ~10-mm steel rod and positioned over each cage. The frame was constructed and positioned so that the cage was shaded throughout the day, including early in the morning when the sun was just above the horizon. The control (i.e., full sun) treatment had a frame placed over each cage, but without any shadecloth. The two shaded treatments each had one of two grades of shadecloth attached to the frames. The solar radiation passing through each grade was measured using the weather station’s sensor on a typical sunny day early in the study, in order to compare SR levels in the cages to those in unshaded cages. Elephant shrews in each of these treatments experienced SR reduced by 78.7% (hereafter, deep shade) and 60.2% (hereafter, partial shade), compared to that experienced by individuals in the full sun treatment. Animals were maintained in the outdoor cages for a period of approximately two months, after which time the iButtons were surgically explanted and *T*_b_ data downloaded. The experiment was conducted in mid-winter (data collection from 15 June–27 July 2013) so as to coincide with the pronounced use of heterothermy by *E*. *myurus* at this time of year.

### Measurements of metabolic heat production capacity

Following the end of the 2-month experimental manipulation of solar radiation and subsequent removal of the iButtons, the elephant shrews were maintained in the outdoor cages for a further two months (August and September 2013). During this time, individuals that had earlier been in the partial shade treatment were allocated randomly to either the full sun or deep shade treatments, with this aspect of the study involving just two treatments instead of three. Following the 2-month acclimation period, BMR, NST capacity and M_sum_ were measured in each individual (full sun n = 6, deep shade n = 8). The order in which these variables were measured in each individual was randomized.

We estimated the BMR of the elephant shrews from rates of oxygen consumption ${\dot{\text{V}}}_{{\text{O}}_{\text{2}}}$ and carbon dioxide production ${\dot{\text{V}}}_{{\text{CO}}_{\text{2}}}$ measured using the same respirometry system as described by Minnaar et al. [18], with animals placed individually in 2-L airtight plastic containers (Lock and Lock, Blacktown, NSW, Australia), modified by adding inlet and outlet ports on opposite sides of each chamber. Dry, CO_2_-free air was pushed through each chamber at 0.7–2.1 L min^-1^, before being sub-sampled downstream of the chamber for measurements of O_2_, CO_2_ and water vapour partial pressures. Mass flow controllers and all analysers were calibrated as described by Minnaar et al. [18]. Before BMR measurements, the elephant shrews’ thermoneutral zone (TNZ) was determined by measuring resting metabolic rate (RMR) and *T*_b_ in six individuals at *T*_a_ = 20, 25, 28, 30, 33 and 36°C. Each *T*_a_ value was experienced by each individual for a minimum of 6 hr and the sequence of *T*_a_ values randomised. A two-segment linear regression model was fitted to RMR *versus T*_a_ data, with the inflection point taken to represent the lower critical limit of thermoneutrality. BMR was measured at *T*_a_ = 30°C as this fell within the TNZ. BMR was measured in two elephant shrews simultaneously, using a respirometry multiplexer (T-RM8, Sable Systems) to subsample air successively from a baseline channel (10 min), followed by the first chamber and then the second (20 min each), and then an additional baseline reading (10 min), with the latter cycle repeated for a minimum of 8 hr, in order to ensure that basal levels were reached [21]. Body mass was measured both before and after BMR measurements, with the average of the two values used for metabolic rate calculations.

Previous studies have demonstrated that *E*. *myurus* shows a strong thermogenic reaction to noradrenalin (NA) injection, which is typically an indication of NST moderated by brown adipose tissue (BAT; [22]). In order to quantify NST capacity we examined the difference between NA-induced metabolic response and RMR. Each elephant shrew received a subcutaneous injection of NA, with the dosage calculated according to the mass of the individual ([23], NA dose (mg/kg] = 2.53 M^0.4^, where M is body mass]. The same respirometry protocol was followed as for BMR with the following modifications. Both RMR and NA-induced metabolic response were measured at 25°C to avoid hyperthermia [24]. Animals were placed in respirometry chambers 2 hr before measurements commenced to allow habituation. Following Mzilikazi and Lovegrove [24], for each individual’s RMR was measured for two hours prior to NA or saline injection. Following the injection, metabolic measurements continued for 1.5 hr or until metabolic rate had returned to RMR. Saline (0.90% NaCl; SAL] was used for control injections to account for the effects of stress associated with handling and injection, with the order of injections (NA or SAL] randomised. Both the control and NA injections for two individuals took place on the same day, by alternating injections between the two individuals. A respirometry multiplexer (T-RM8, Sable Systems] was used to subsample air and was switched between the two chambers manually as required. Recordings followed a general pattern of baseline (5 min], RMR (2 hr], injection (1.5 hr] and a final baseline (5 min] for each injection.

We measured *M*_sum_ in the elephant shrews in a helox (21% O_2_, 79% He] atmosphere, using a sliding cold exposure protocol [25] and the same setup as Minnaar et al. [18]. Elephant shrews were placed individually in a 2-L chamber. To measure *T*_a_, a calibrated Cu-Cn thermocouple was inserted through a small, grommet-sealed hole in the chamber wall. Atmospheric air was supplied to each chamber at a flow rate of 1.5 L min^-1^ for approximately 10 min after an elephant shrew was placed in the chamber. Thereafter, the atmospheric air was replaced by helox at the same flow rate. During this time, chamber temperature was kept at room temperature, as lower temperatures caused elephant shrews to enter torpor. Sufficient time was allowed for helox to replace atmospheric air in the chamber whilst simultaneously recording a helox baseline (5–10 min). Thereafter, the sliding cold exposure protocol was initiated by reducing the temperature setpoint of the fridge/freezer to the minimum value (-18°C) which resulted in a chamber cooling rate of ~10°C hr^-1^. Measurements continued until ${\dot{\text{V}}}_{{\text{O}}_{\text{2}}}$ reached a plateau and ceased increasing with decreasing *T*_a_. To verify that *M*_sum_ had in fact been elicited, *T*_b_ was measured immediately upon each animal’s removal from the chamber using a handheld PIT tag scanner (DTR-4, Destron Fearing, St Paul, Minnesota, USA) to ensure that the elephant shrew had become hypothermic and hence that maximum heat production capacity was attained. A final baseline reading (5–10 min) was then taken by flowing helox through the analysers. Mean respiratory exchange ratio (RER) during *M*_sum_ measurements was 0.665 ± 0.054.

### Data analyses

All values are presented as mean ± SD for *n* = number of individuals and *N* = number of observations. Due to the controversy surrounding the ability to distinguish downregulated metabolic rate and *T*_b_ from normothermic levels [26–30], departures from normothermy were determined by fitting a normal distribution curve to all *T*_b_ ≥ the upper modal *T*_b_, following McKechnie et al. [31]. The lower 99% confidence interval was estimated for each individual’s normothermic *T*_b_ distribution, with the upper threshold *T*_b_ for torpor defined by subtracting 3°C from the lower 99% confidence interval of the *T*_b_ distribution [31]. Torpor bout duration (min) was thus calculated as the total time *T*_b_ was maintained below the estimated threshold for each individual, and the time of torpor entry and torpor arousal determined by taking the time where *T*_b_ initially decreased below the threshold and when it returned to the threshold value, respectively. Minimum *T*_b_ (T_b-min_) was taken as the lowest *T*_b_ value measured during a 24-hr period. Rewarming rate (°C min^-1^) for each bout was taken as the slope of a linear regression model fitted to all *T*_b_ values between the onset of arousal and the attainment of normothermic *T*_b_, using Expedata software (Sable Systems, Las Vegas NV). The extent of heterothermy for each elephant shrew was also quantified using the Heterothermy Index (HI; [30]). The HI quantifies variation in *T*_b_ around the modal normothermic *T*_b_, and is calculated as

$$HI\;=\;\sqrt{\frac{\sum {\left(T_{b-mod}-T_{b-i}\right)}^{2}}{n-1}}$$

where *T*_b-mod_ is the modal normothermic *T*_b_ of an individual, *T*_b,i_ is the *T*_b_ at time *i*, and n is the number of times *T*_b_ is sampled [30]. Finally, torpor frequency was calculated as the percentage of days on which torpor was used during the study period. Days on which cloud cover was present in the morning were excluded from the analyses. To compare torpor characteristics between the three experimental treatments, relationships between response variables (T_b-min,_ HI, rewarming rate, torpor bout duration, time of rewarming or torpor frequency) and predictor variables (T_a_, treatment, sex and mass) were examined using generalized linear mixed models (GLMM) when data distribution was non-normal, and linear mixed models (LMM) when normality assumptions were met. All analyses were conducted using R 3.0.2 (R Foundation for Statistical Computing 2013). Assumptions concerning normality were verified using qq plots and Shapiro-Wilk tests, and homoscedasticity was confirmed using Levene’s tests. To evaluate between-treatment differences in torpor characteristics, models were run using the *lme4* [32] and *mvtnorm* [33] packages as applicable. Sex was initially included as a predictor, but as it was not significant was dropped from further analyses of torpor variables.

Differences in BMR, NST and *M*_sum_ between the two treatments used for investigating phenotypic flexibility in heat production capacity were evaluated by fitting linear mixed models using the *nlme* package [34]. BMR, *M*_sum_, ME, thermogenic capacity or saline-induced MR were used as response variables, and treatment, injection type, sex and *M*_b_ as predictors. Interactions between predictor variables were not significant and were thus dropped from further analyses. Body mass was modeled using treatment and sex as fixed effects and individual as a random effect. There were no significant differences in *M*_b_ between treatments or sexes, and so data for males and females were pooled during subsequent analyses. Thermogenic capacity was also analysed using linear mixed models with the same general structure, as was the difference in injection-induced metabolic response between control and NA injections. In addition, student *t*-tests were used to compare differences between BMR and NA-injection induced MR as well as to compare BMR and *M*_sum_.

## Results

### Experimental manipulation of solar radiation availability

The elephant shrews’ *M*_b_ remained approximately stable throughout the experiments. The mean *M*_b_ was 52.1 ± 5.4 g (females, *n* = 9) and 50.7 ± 4.0 g (males, *n* = 9) prior to the experiment, and 52.2 ± 5.0 g and 54.7 ± 4.5 g, respectively, at the end. Male and female *M*_b_ did not differ significantly before (LMM, *F*_1,16_ = 0.409, *P* = 0.531) or after (LMM, *F*_1,16_ = 2.188, *P* = 0.157) the experiments.

Typical basking behaviour, where the body was flattened against the rock substrate perpendicular to the sun’s rays, was observed exclusively in the unshaded treatment. This behaviour took place from approximately 07h00 onwards. Animals appeared to bask throughout the day, moving intermittently to shaded spots during the heat of the day. Individuals in the shaded treatments were occasionally observed pressing their bodies flat to the ground away from the rock pile, but it was unclear whether this was basking behaviour or sand-bathing.

**Fig 1:**
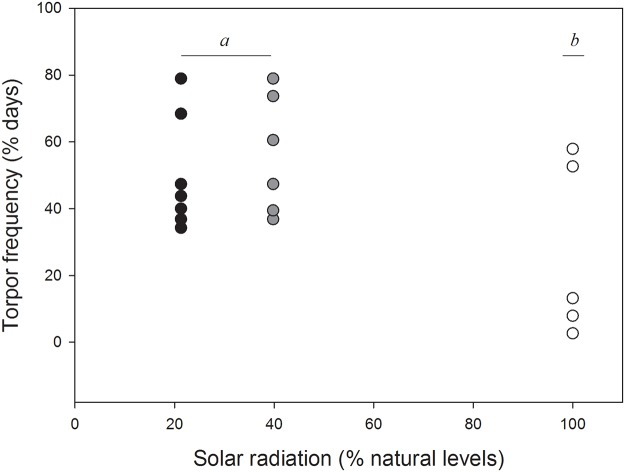
Frequency of torpor. Percentage of days on which torpor was expressed in eastern rock elephant shrews (*Elephantulus myurus*) housed in outdoor cages with exposure to natural (100%) or experimentally reduced (approximately 20% or 40%) levels of solar radiation. Each data point represents one individual.

A total of 297 torpor bouts were recorded over 36 days. Mean torpor frequency (i.e., percentage of days on which torpor was used) was 58.3 ± 18.1% (N = 122 bouts) in the deep shade treatment, 54.8 ± 20.3% (N = 124 bouts) in the partial shade treatment, and 27.8 ± 28.1% (N = 51 bouts) in the full sun treatment, with variation among the three treatments being marginally non-significant (LMM, *F*_1, 16_ = 3.98, *P* = 0.06). However, when data were pooled for the partial and deep shade treatments, individuals that received shading used torpor significantly more frequently than unshaded animals (LMM, *F*_1,16_ = 5.33, *P* = 0.035, Fig. 1). Minimum torpor *T*_b_ did not vary among treatments (GLMM, *t* = 0.386, *P* = 0.699), with an overall average of 15.3 ± 6.8°C. The lowest *T*_b_ value recorded during the experiment was 8.9°C.

**Fig 2:**
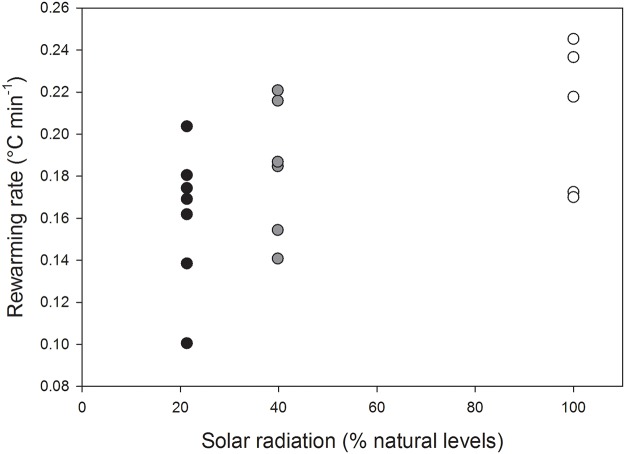
Rewarming rate during arousal. Eastern rock elephant shrews (*Elephantulus myurus*) housed in outdoor cages with exposure to natural (100%) or experimentally reduced (approximately 20% or 40%) levels of solar radiation. Each data point represents one individual.

Rewarming rates increased significantly with access to solar radiation (LMM, *t* = 2.150, *P* = 0.047) with mean values of 0.15 ± 0.05°C min^-1^, 0.18 ± 0.06°C min^-1^ and 0.20 ± 0.05°C min^-1^ for the deep shade, partial shade and full sun treatments, respectively (Fig. 2).

Most entries into torpor occurred at night. However, the time of torpor entry appeared to be bimodally distributed, with an initial peak between 19h00–00h00 and a second peak just before sunrise. The time at which rewarming commenced ranged from 22h00 to 08h00, with most rewarming commencing between 05h00 and 08h00. The time at which rewarming commenced (expressed as minutes before sunrise), did not vary significantly among treatments (GLMM, *t* = 0.890, *P* = 0.698). In all three treatments, partial rewarming generally occurred before sunrise. Torpor bout duration also did not vary among treatments (GLMM, *t* = 0.977, *P* = 0.349), with a pooled mean of 463 ± 220 min.

**Fig 3:**
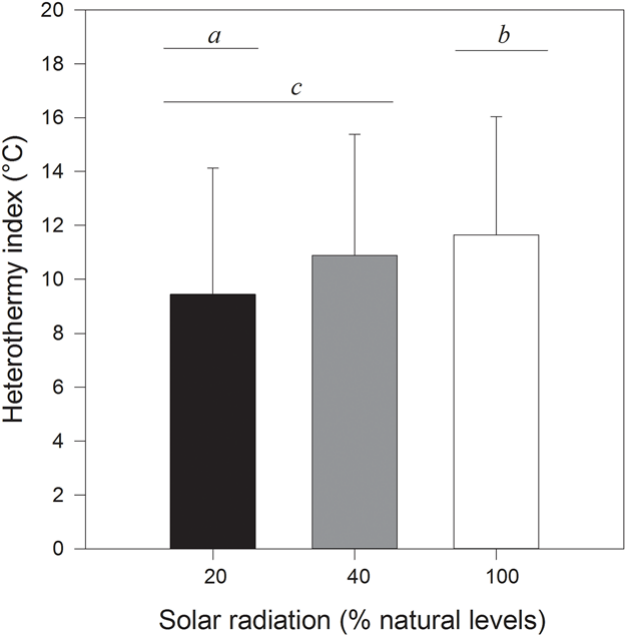
Overall mean heterothermy index [30]. Eastern rock elephant shrews (*Elephantulus myurus*) housed in outdoor cages with exposure to natural (100%) or experimentally reduced (approximately 20% or 40%) levels of solar radiation.

HI values varied significantly among treatments (GLMM, *t* = 2.56, *P* = 0.01), with HI values of 9.43 ± 4.69°C, 10.89 ± 4.49°C and 11.65 ± 4.38°C for the deep shade, partial shade and full sun treatments, respectively (Fig. 3). Significant differences existed between full sun and deep shade treatments (Tukey HSD, *P* = 0.012) as well as between partial shade and deep shade (Tukey HSD, *P* = 0.015).

### Phenotypic flexibility in heat production capacity

**Fig 4:**
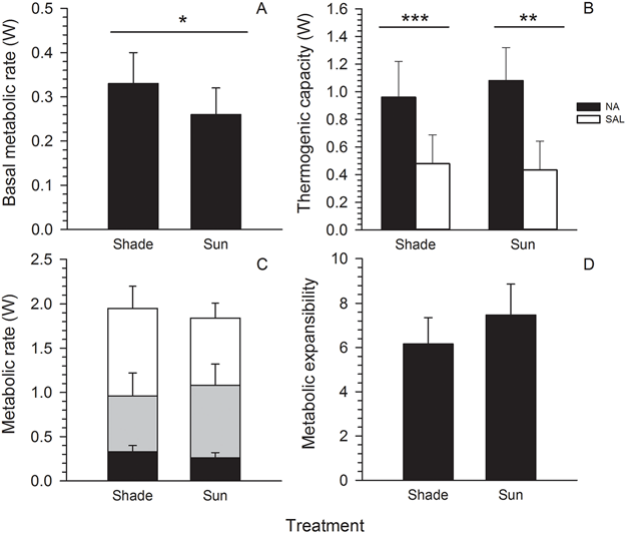
Metabolic rates. Basal metabolic rate (BMR), non-shivering thermogenesis (NST) capacity and summit metabolism (*M*_sum_) in eastern rock elephant shrews (*Elephantulus myurus*) housed in outdoor cages with exposure to natural (100%, “Sun”) or experimentally reduced (approximately 20%, “Shade”) levels of solar radiation. Panel A: BMR; panel B: metabolic rate induced by either noradrenalin (NA) or a control saline (SAL) injection during measurements of NST, panel C: BMR (black), NST capacity (grey) and *M*_sum_ (white); panel D: metabolic expansibility (i.e., *M*_sum_ / BMR).

The BMR of elephant shrews maintained in full sun (0.26 ± 0.06 W, *n* = 6) was significantly lower compared to that of individuals housed in deep shade (0.33 ± 0.07 W, *n* = 8; LMM, F_1, 13_ = 4.808, *P* = 0.047; Fig. 4A). When body mass was included in the analysis, this difference was statistically even more pronounced (LMM, F_1,14_ = 6.113, *P* = 0.028). Neither BMR (LMM, F_1,14_ = 0.056, *P* = 0.816), nor mass (LMM, F_1,14_ = 0.432, *P* = 0.522) differed between sexes.

Injection of NA caused an easily observable increase in metabolic rate. NA-induced metabolic rate varied significantly with sex, with males showing greater responses (mean = 1.22 ± 0.12 W) than females (mean = 0.93 ± 0.23 W; LMM, F_1,5_ = 8.130, *P* = 0.04). The difference in metabolic rate between BMR and post-NA injection was significant in both shaded (*n* = 8; t-test, t = -7.098, *P* < 0.001) and unshaded (*n* = 6; t-test, t = -7.494, *P* < 0.001) treatments. The increase in metabolic rate between BMR and post-NA injection also differed significantly between the deep shade and full sun treatments (LMM, F_1,10_ = 10.249, *P* = 0.01). The metabolic rate of individuals in the shaded treatment increased to 2.96 ± 0.66 X BMR after NA injection, whereas full sun animals showed a significantly higher increase to 4.02 ± 1.02 X BMR (Fig. 4C). The increase in metabolic rate induced by the control SAL injection was significantly lower than that of NA-induced metabolic rate for both the shaded (means = 0.48 ± 0.20 W, 0.96 ± 0.24 W respectively) and unshaded (means = 0.43 ± 0.21 W, 1.08 ± 0.26 W respectively) treatments (LMM, F_1,13_ = 51.209, *P* < 0.001). However, NST capacity (i.e, the difference between NA-induced metabolic rate and SAL-induced metabolic rate) did not differ between the two treatments (LMM, F_1,11_ = 1.257, *P* = 0.286), and we thus conclude that treatment did not influence overall NST capacity.

Mean mass-specific *M*_sum_ values were 35.40 ± 5.03 mW g^-1^ in deep shade and 32.96 ± 3.37 mW g^-1^ in full sun, and did not differ significantly (LMM, F_1,13_ = 1.404, *P* = 0.257). Whole-animal *M*_sum_ values were also not significantly different between shaded (1.95 ± 0.25 W) and unshaded treatments (1.84 ± 0.17 W; LMM, F_1,13_ = 1.119, *P* = 0.309; Fig. 4C). As was the case for BMR, *M*_sum_ did not vary significantly with sex (LMM, F_1,14_ = 2.636, *P* = 0.127).

Overall ME values did not differ between shaded (mean = 6.16 ± 1.40) and unshaded (mean = 7.47 ± 1.91) treatments (LMM, F_1,13_ = 2.293, *P* = 0.154; Fig. 4D), nor did mass-specific ME values (LMM, F_1,13_ = 4.191, *P* = 0.067), although the latter difference approached significance (full sun: 7.60 ± 1.74 *vs* shade: 6.15 ± 1.11). The ME of animals in the shaded treatment ranged from 4–8, whilst the full sun treatment had a broader range of 5–11. As was the case for BMR and *M*_sum_, there was no significant effect of sex on ME (LMM, F_1,14_ = 1.341, *P* = 0.266).

## Discussion

Artificial reductions in solar radiation availability affected patterns of heterothermy in *E*. *myurus* housed in outdoor enclosures in several ways. Rewarming rates were positively related to solar radiation availability, with animals in unshaded cages rewarming more rapidly than individuals in partially shaded cages. Furthermore, torpor bouts were less frequent, but overall levels of heterothermy higher, in elephant shrews experiencing natural solar radiation availability compared to those in shaded treatments.

As was the case for wild conspecifics [3], captive elephants shrews in our study typically completed rewarming within 2–3 hr following sunrise. The range of rewarming rates we observed (individual means of approximately 0.10–0.25°C min^-1^) was narrower than that exhibited by the free-ranging population studied by Mzilikazi et al. [3]. In the latter study, free-ranging *E*. *myurus* rewarmed at rates of anywhere between 0.01 and 0.35°C min^-1^, with inferred SR-assisted rates between 0.01 and 0.10°C min^-1^ and inferred endogenous (i.e., not associated with basking behaviour) rates between 0.25 and 0.35°C min^-1^. Mzilikazi et al. [3] did not directly observe SR-assisted rewarming, but inferred it from comparisons of observed rewarming rates of elephant shrews to those of blackbody probes. In contrast, we directly observed elephant shrews basking.

In terms of evaluating the functional significance of basking in *E*. *myurus*, a key finding of our study is that rewarming rates were higher in elephant shrews with access to natural solar radiation levels. Taken together with our observations of elephant shrews basking while rewarming, the higher rewarming rates of individuals in the unshaded treatment reveals that the elephant shrews used solar radiation to supplement, rather than substitute, endogenous metabolic thermogenesis. McKechnie and Wolf [11] modelled the energetic consequences of basking during rewarming, and noted that passive rewarming (i.e., rewarming during which solar radiation entirely replaces endogenous thermogenesis), will usually involve substantially lower rewarming rates than those associated with endogenous rewarming. In the case of *E*. *myurus*, this prediction is consistent with the much lower rewarming rates of individuals inferred to be rewarming passively compared to those rewarming using endogenous heat sources [3], and there is similar evidence from a range of other mammalian and avian species [reviewed by McKechnie and Wolf [11]].

Comparisons of rewarming rates observed in the present study and those observed by Mzilikazi et al. [3] also suggest that our captive *E*. *myurus* used a different mode of SR-assisted rewarming compared to wild conspecifics. In Mzilikazi et al.’s study, individuals inferred to be basking rewarmed far more slowly than animals rewarming endogenously, whereas the opposite was true in the present study. Rewarming rates associated with basking in individuals in the unshaded treatment (mean = 0.20°C min^-1^) were well above the rewarming rates associated with inferred basking behaviour (< 0.1°C min^-1^) in free-ranging individuals [3]. However, these mean rates were also slightly below the range of endogenous rewarming rates documented by the latter authors (0.25–0.35°C min^-1^), although the maximum rewarming rate observed during the present study (0.34°C min^-1^) is virtually identical to the maximum rates in wild conspecifics. Thus, *E*. *myurus* in all three treatments in the present study showed mean rewarming rates intermediate between the passive and endogenous rates inferred for wild individuals.

A potential explanation for this pattern is that, in all three treatments, elephant shrews showed approximately equal endogenous rewarming rates, and the increase in rewarming rate with SR availability simply reflects the additive effect of SR-driven warming during basking. This possibility can be evaluated using the solar irradiance data collected using the weather station, and estimating the range of associated passive rewarming rates. During the study period, solar irradiance between 07h00 and 09h00 ranged from 10–340 W m^-2^. The theoretical maximum passive rewarming rate (i.e., the rate at which an animal’s tissues will warm when heat loss to the environment is zero) can be calculated as $R_{P_{\text{max}}}$ = SHG/(s*M*_b_), where $R_{P_{\text{max}}}$is the maximum passive rewarming rate (°C min^-1^), SHG is solar heat gain (J min^–1^), s is the specific heat of animal tissues (3.43 J g^-1^°C^-1^), and *M*_b_ is body mass (g) [11]. In small mammals, the percentage of intercepted solar radiation that contributes to heat balance is typically around 20% [35–37], but may be as high as ~ 60% [38, 39]. Based on average solar radiation between 07h00 and 09h00 during our study (161 W m^-2^) and an average surface area of the elephant shrews of 74.7 cm^2^ [mean for 10 individuals, calculated using the same approach as Marom et al. [40]], and assuming that the projected surface that intercepts solar radiation during rewarming is equivalent to 30% of SA, estimated $R_{P_{\text{max}}}$ values range from 0.024°C min^-1^ (20% of solar radiation absorbed) to 0.072°C min^-1^ (60% of solar radiation absorbed). The observed difference in rewarming rates between the deep shade and full sun treatments (~ 0.05°C min^-1^) falls within this range, supporting the notion that the observed difference between the treatments reflects the additive effect of solar radiation on the heat balance of the individuals in the full sun treatment.

One potential albeit highly speculative explanation for why elephant shrews in this study did not show the very low rewarming rates associated with inferred periods of passive rewarming in wild conspecifics [3] relates to perceived predation risk. Two potential diurnal predators, namely black-headed heron (*Ardea melanocephala*) and yellow mongoose (*Cynictis penicillata*), were observed near the outdoor cages during the study, and their presence may have contributed to the initiation of rewarming prior to sunrise. According to this idea, elephant shrews with access to natural levels of solar radiation in our study rewarmed far more rapidly than conspecifics in Mzilikazi et al.’s [3] study on account of the presence of potential predators. An interesting avenue for future work will be to experimentally investigate whether the functional role of solar radiation during rewarming varies with perceived predation risk.

Other parameters of torpor bouts in the present study also overlapped with values observed in wild conspecifics. Torpor bout duration was more variable, ranging from 0.5–14.3 h, compared to 1–27 hr in free-ranging *E*. *myurus* [3], indicating that under semi-natural conditions torpor bout lengths were shorter overall. Moreover, torpor frequency under semi-natural conditions varied more than in wild individuals. Individuals in the present study used torpor on 3–61% of days in the unshaded treatment, 39–83% in partial shade and 36–78% in deep shade, whereas the corresponding values for wild conspecifics ranged from 19–64% [3].

In addition to significant variation in rewarming rates and other parameters of torpor, artificial manipulation of solar radiation also resulted in metabolic adjustments in *E*. *myurus*. The BMR of individuals in shaded cages was higher compared to that of conspecifics with access to natural levels of solar radiation. In contrast, both NST capacity and *M*_sum_ were statistically indistinguishable between the two treatments.

The higher BMR in the shaded treatment is qualitatively similar to metabolic changes induced by cold acclimation among small mammals in general [22, 41–44]. Increases in BMR in response to thermal acclimation are often associated with an extension of the thermoneutral zone to include lower *T*_a_s [41] as well as enhancements of thermogenic capacity [22, 44]. However, it is noteworthy that in the present study these changes were not associated with differences in *T*_a_ among treatments, but rather variation in the availability of solar radiation. Our data thus provide further evidence that mammalian BMR is highly responsive to short-term changes in thermal environments, even if these changes are independent of *T*_a_, and reiterate the significance of solar radiation in determining the microclimates experienced by small mammals and the close links between those microclimates and their thermal physiology.

The BMR values we report here are lower than values previously reported for *E*. *myurus*. The mean BMRs in the present study (unshaded: 0.26 W; shaded: 0.33 W) compare with means of 0.42 W and 0.56 W for warm- and cold-acclimated individuals, respectively, reported by Mzilikazi et al. [3]. A BMR of 0.38 W, closer to our observed value, was reported for *E*. *myurus* maintained at *T*_a_ = 18°C [45]. The lower BMR in *E*. *myurus* housed under natural conditions of *T*_a_ and photoperiod contrasts with the mammalian pattern of the BMR of populations housed under natural conditions generally being higher than conspecifics maintained under constant environmental temperatures [46]. It must, however, be noted that the comparison of among-study variation in the BMR in *E*.*myurus* involves populations occurring in different regions of South Africa, with different climates.

The NST capacity in the present study was slightly higher than previously reported for *E*. *myurus*. Mzilikazi et al. [22] reported NST capacity of 0.94 ± 0.24 W for warm-acclimated and 0.91 ± 0.22 W for cold-acclimated individuals (values converted to W assuming RER = 0.85), whereas NA-induced values in the present study averaged 1.01± 0.25 W (pooled data for both treatments). The sex difference in NA-induced MR was unexpected. Sexual dimorphism in thermogenic capacity usually involves higher thermogenic activity in BAT tissue of females compared to males [47]. In contrast, NA-induced MR was higher in male *E*. *myurus*, although the small sample size for males means that this conclusion needs to be verified.

The lack of a significant difference in *M*_sum_ and ME between the two treatments reveals that heat production capacity was not adjusted in response to reduced availability of solar radiation. Interspecific variation in ME has been correlated with rewarming in rodents, with species rewarming in colder environments and from lower heterothermic *T*_b_ having higher ME [19]. It has also recently been suggested that M_sum_ may increase within individuals when thermogenesis associated with activity is curtailed through captivity [18].

To the best of our knowledge, *M*_sum_ has not been measured in any other afrotherian mammal [48]. As was the case in Wahlberg’s epauletted fruit bats (*Epomophorus wahlbergi*) [18], RER values during *M*_sum_ measurements in *E*. *myurus* fell below the theoretically expected range of 0.71–1.00, further reiterating the need for further investigation of the substrates metabolised by mammals during acute cold stress. The mean *M*_sum_ we observed in this study is close to the value predicted for a 56-g mammal, being equivalent to 88% of the value predicting using the conventional (i.e., non-phylogenetically-corrected) allometric equation presented by Minnaar et al. [18]. Moreover, the ME of *E*. *myurus* falls within the typical mammalian range of 4–8 [19, 48].

In conclusion, the combination of a) more rapid rewarming in elephant shrews with access to natural levels of solar radiation compared to those in treatments where radiation was artificially reduced, and b) the lack of phenotypic adjustments in NST capacity and M_sum_, suggests that under the conditions of our study solar radiation served to supplement, rather than substitute, endogenous metabolic thermogenesis during rewarming from torpor. However, these findings should be seen in the context of this study involving captive individuals held in cages with a predictable food supply. The ways in which solar radiation interacts with endogenous metabolic thermogenesis in free-ranging individuals in natural habitats may be quite different, and may vary with factors such as fluctuating food availability and predation risk.
